# Policy Compression for Intelligent Continuous Control on Low-Power Edge Devices

**DOI:** 10.3390/s24154876

**Published:** 2024-07-27

**Authors:** Thomas Avé, Tom De Schepper, Kevin Mets

**Affiliations:** 1 IDLab—Department of Computer Science, University of Antwerp—IMEC, Sint-Pietersvliet 7, 2000 Antwerp, Belgium; thomas.ave@uantwerpen.be; 2 AI & Data Department, IMEC, 3001 Leuven, Belgium; tom.deschepper@imec.be; 3 IDLab—Faculty of Applied Engineering, University of Antwerp—IMEC, Sint-Pietersvliet 7, 2000 Antwerp, Belgium

**Keywords:** policy distillation, model compression, DRL, continuous action spaces, soft actor-critic, edge computing

## Abstract

Interest in deploying deep reinforcement learning (DRL) models on low-power edge devices, such as Autonomous Mobile Robots (AMRs) and Internet of Things (IoT) devices, has seen a significant rise due to the potential of performing real-time inference by eliminating the latency and reliability issues incurred from wireless communication and the privacy benefits of processing data locally. Deploying such energy-intensive models on power-constrained devices is not always feasible, however, which has led to the development of model compression techniques that can reduce the size and computational complexity of DRL policies. Policy distillation, the most popular of these methods, can be used to first lower the number of network parameters by transferring the behavior of a large teacher network to a smaller student model before deploying these students at the edge. This works well with deterministic policies that operate using discrete actions. However, many real-world tasks that are power constrained, such as in the field of robotics, are formulated using continuous action spaces, which are not supported. In this work, we improve the policy distillation method to support the compression of DRL models designed to solve these continuous control tasks, with an emphasis on maintaining the stochastic nature of continuous DRL algorithms. Experiments show that our methods can be used effectively to compress such policies up to 750% while maintaining or even exceeding their teacher’s performance by up to 41% in solving two popular continuous control tasks.

## 1. Introduction

Deep reinforcement learning (DRL) methods have been shown to be highly effective at solving discrete tasks in constrained environments, such as energy-aware task scheduling [1] and offloading [2,3] in edge networks, 5G beamforming and power control [4], and network function (NF) replica scaling [5] in software-defined networking (SDN). These tasks can be solved by performing a sequence of actions that are chosen from a discrete set, such as whether to offload a task or process it locally. However, many task solutions cannot be effectively decomposed in such a way, such as fluid movement in robotics pathfinding that allows precise control [6], the continuous control of drone steering [7], the amount of resources to allocate in micro-grids [8], and multi-beam satellite communication [9]. These types of problems are referred to as continuous control tasks.

In reinforcement learning, the action space refers to the set of possible actions an agent can take in a given environment. Different types of DRL algorithms have been developed that can learn this type of behavior by working with continuous action spaces. In contrast to discrete action spaces, that take the form of a limited (usually fixed) set of actions to choose from, these algorithms can perform actions using real-valued numbers, such as the distance to move or how much torque to apply. This distinction has significant implications for the types of tasks and the models used to solve them.

Discrete actions are best suited for tasks that involve some form of decision-making in an environment with less complex dynamics. Take for example a wireless edge device that optimizes for a stable connection while roaming between several access points (APs). When modeled as a discrete task, the agent can decide at each time step to which from a set of known APs it should connect. This might not always be the one with the strongest signal, as the predicted path of the agent could align better with another AP. There are other ways, however, to optimize a connection with an AP before having to initiate handover that require more granular control, such as adjusting the transmission power and data rates. Such problems, which require continuous control and parameter optimization, are best modeled using continuous action spaces.

Learning continuous behaviors is often more challenging than learning discrete actions, however, as the range of possible actions to explore before converging on an optimal policy is infinite. A common approach is, therefore, to discretize the continuous actions into a fixed set of possible values [4], but this can lead to a loss of accuracy when using a large step size or drastically increase the action space and therefore learning complexity [10]. It also removes any inherent connection between values that are close to each other, making it more difficult to converge to an optimal policy. Another solution is to learn a policy that samples actions from a highly stochastic continuous distribution, which can increase robustness and promote intelligent exploration of the environment. This method is employed by the Soft Actor-Critic (SAC) algorithm [11] for example.

Since many tasks are carried out on battery-powered mobile platforms, additional constraints apply in terms of computing resources and power consumption. This can make it practically infeasible to deploy large models on low-power edge devices that need to perform such tasks. Some methods have been introduced to combat this, by reducing the size and therefore computational complexity of the deep neural networks (DNNs) through which the DRL agent chooses its actions, without decreasing its effectiveness at solving the task. In DRL, one of the most popular model compression techniques is policy distillation [12]. Here, a Deep Q-Network (DQN) can be compressed by transferring the knowledge of a larger teacher network to a student with fewer parameters. Compressing DRL models enables low-power devices to perform inference using these models on the edge, increasing their applicability, reducing cost, enabling real-time execution, and providing more privacy. These benefits have recently been demonstrated in the context of communication systems and networks for the compression of DRL policies that dynamically scale NF replicas in software-based network architectures [5].

However, the original policy distillation [12] method was only designed for policies from DQN teachers, which can only perform discrete actions. Most subsequent research has also continued in the same direction by improving distillation for teachers with discrete action spaces [5,13,14,15]. DQNs are also fully deterministic, meaning that, for a given observation of the environment, they will always choose the same action. But policies for continuous action spaces are generally stochastic, by predicting a distribution from which actions are sampled. In this paper, we therefore:

1. Propose three loss functions that allow for the distillation of continuous actions, with a focus on preserving the stochastic nature of the original policies.
2. Highlight the difference in effectiveness between the methods depending on the policy stochasticity by comparing the average return and action distribution entropy during the evaluation of the student models.
3. Provide an analysis of the impact of using a stochastic student-driven control policy instead of a traditional teacher-driven approach while gathering training data to fill the replay memory.
4. Measure the compression potential of these methods using ten different student sizes, ranging from 0.6% to 100% of the teacher size.
5. Benchmark these architectures on a wide range of low-power and high-power devices to measure the real-world benefit in inference throughput of our methods.

We evaluate our methods using an SAC [11] and PPO [16] teacher on the popular HalfCheetah and Ant continuous control tasks [17]. Through these benchmarks, in which the agent needs to control a robot with multi-joint dynamics, we focus on an autonomous mobile robotics use case as a representative example of a power-constrained stochastic continuous control task. However, our methods can be applied to any DRL task defined with continuous action spaces, including the previously mentioned resource allocation tasks.

These experiments demonstrate that we can effectively transfer the distribution from which the continuous actions are sampled, thereby accurately maintaining the stochasticity of the teacher. We also show that using such a stochastic student as a control policy while collecting training data from the teacher is even more beneficial, as this allows the student to explore more of the state space according to its policy, further reducing the distribution shift between training and real-world usage. Combined, this led to faster convergence during training and better performance of the final compressed models.

## 2. Background

### 2.1. Reinforcement Learning

**Figure 1:**
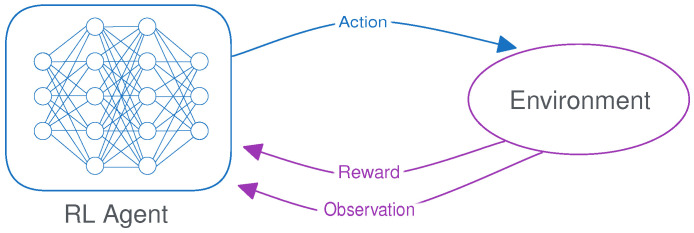
An illustration of the reinforcement learning loop.

Reinforcement learning (RL) is a machine learning technique in which an agent learns a policy through trial and error by interacting with an environment, which is specified using a Markov decision process (MDP). Unlike supervised learning, where models learn from labeled examples, RL agents dynamically perform actions based on an observation of the current state of the environment and receive feedback in the form of a numerical reward signal [18] (as illustrated in Figure 1). By taking this action, the state of the environment is updated to reflect the consequences. This mapping from states to actions is called the policy, and the goal of the agent is to learn a policy that maximizes the (discounted) cumulative reward over an episode, called the return. An episode is a sequence of states, actions, and rewards that starts at the initial state and ends when a terminal state is reached. In DRL, the policy takes the form of a neural network that takes the state as input and outputs the action to be taken. This policy network is optimized using the reward signal to encourage or discourage certain behavior.

**Figure 2:**
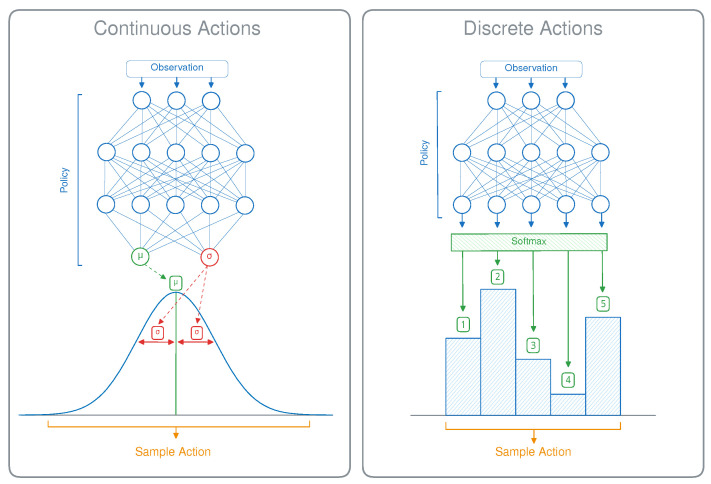
Discrete and continuous action spaces for stochastic DRL policies.

MDPs can work with either discrete or continuous action spaces, depending on the nature of the task. An illustration of both is given in Figure 2. For discrete tasks, the agent can choose from a limited set of options, such as a cardinal direction to move in. In that case, the neural network outputs a single value for each possible action and the policy consists of either choosing the action with the highest value or sampling from the distribution of these values. For continuous tasks, the agent can perform actions using a combination of real-valued numbers, such as the distance to move or how much torque to apply. The goal of this paper is to provide a method that can effectively compress such a policy network for continuous action spaces.

#### Continuous Action Spaces

DRL algorithms for continuous action spaces, such as PPO [16], A2C [19], SAC [11], or TD3 [20], work by modeling the policy as a continuous probability distribution from which actions are sampled. In practice, this almost always takes the form of a normal distribution, as shown in Figure 2, so this will be our focus in this paper. The model then predicts a mean value (μ) for each action, and the actual policy consists of sampling actions based on this mean and a standard deviation (σ). This standard deviation can in effect be used to control the trade-off between exploitation and exploration, using a lower or higher value of σ, respectively. There are several methods for modeling σ, either algorithmically or learned by the model. Depending on the implementation, either a representation is learned that is dependent on the current state of the environment or one that simply consists of a state-independent vector. In the state-dependent setting, the model can learn to increase or decrease exploration for certain parts of the environment, depending on its degree of uncertainty.

For most algorithms and environments, the learned σ should generally gradually decrease during training as the certainty about the environment increases. Often, the deterministic policy that consists of always choosing the predicted mean action (with σ=0) will produce the best results during evaluation [11], but this is not always the case. For environments that are either only partially observable (POMDPs), are non-deterministic, or contain state aliasing, a stochastic policy can be optimal. Since the policy is trained with this stochasticity in place, it is sometimes detrimental to remove it, as the policy has learned to rely on it. This is especially true for SAC agents, which are trained to maximize an entropy-regularized return and, therefore, to obtain the highest possible return while also remaining as stochastic as possible.

### 2.2. Policy Distillation

The concept of knowledge distillation (KD) as a model compression method was first introduced in the context of supervised learning [21] and later extended to deep reinforcement learning (DRL) policies by Rusu et al. [12]. It works by compressing a deep neural network (DNN) that is designated as the teacher and training a smaller student network to emulate the output of the larger teacher. After training both on a more powerful computing instance, the students can be deployed efficiently on low-power edge devices, where the teacher would be too large to run effectively. Training data are collected by recording the observations and the teacher’s network outputs in a replay memory (*D*) while interacting with the environment by choosing actions according to the teacher’s policy. This replay memory is periodically refreshed to widen the distribution of states encountered by the student.

The original policy distillation [12] method was teacher-driven, meaning that the policy of the teacher is followed while collecting transitions to fill *D*. With a student-driven control policy, the actions are chosen by the student while still storing the teacher outputs in *D* [13]. This reduces the distribution shift between the data the student is trained on and what it encounters during testing, compared to the teacher-driven approach. Small inaccuracies of the student policy can be an insignificant contribution to the distillation loss but have a large impact on the task performance when this causes a transition to a part of the state space that is not encountered when only following the optimal trajectories sampled from the teacher policy. By following the student as the control policy instead, these mistakes will also be encountered during training. This increases the distillation loss for those suboptimal transitions and allows the student to learn how the teacher would recover from them. In theory, those errors should not occur when the student is trained to accurately emulate the teacher policy. But especially in the context of model compression, where the students are only a fraction of the size of their teacher, this is a difficult objective to achieve without overfitting. For some environments and teachers, this distribution shift is more pronounced than for others, making the student-driven approach not necessarily automatically the best choice. Sometimes, it can also lead to slower convergence because the first collected trajectories are suboptimal, and it is more expensive during training since the network outputs of both the teacher and the student are needed for each transition during data collection.

Instead of directly training a smaller network, the student only needs to learn how to follow the final teacher policy, while the teacher still contains redundant exploration knowledge about suboptimal trajectories [12]. This knowledge is necessary to find the optimal policy but not to follow it, so it can be omitted from the student. In student-driven distillation, the student also learns more exploration knowledge, but in practice, it will still follow the final teacher policy relatively closely. Using overcomplete DRL models also helps with alleviating optimization issues, such as becoming stuck in local minima, which occur less when learning to emulate an existing network in distillation [12].

Policy distillation [12] distinguishes itself from imitation learning by not simply learning the best action given a state of the environment but also valuable secondary ‘dark’ knowledge that is expressed in all the teacher network outputs [22]. Since policy distillation [12] was originally developed for DQN teachers, the other network outputs correspond to the state-action (Q) values for all possible discrete actions. These Q-values represent the expected (discounted) return when taking that particular action in the current state, with the highest value indicating the best action. The student is trained using the Kullback–Leibler divergence (KL) between the teacher ($q^{T}$) and the student ($q^{S}$) outputs, with $\theta_{S}$ the trainable student parameters and τ a temperature used to sharpen or smoothen the teacher outputs:

(1) $$L_{KL}\left(D,\theta_{S}\right)=\sum_{i=1}^{\left|D\right|}\mathrm{softmax}\left(\frac{q_{i}^{T}}{\tau }\right)\ln\left(\frac{\mathrm{softmax}(\frac{q_{i}^{T}}{\tau })}{\mathrm{softmax}(q_{i}^{S})}\right)$$

**Figure 3:**
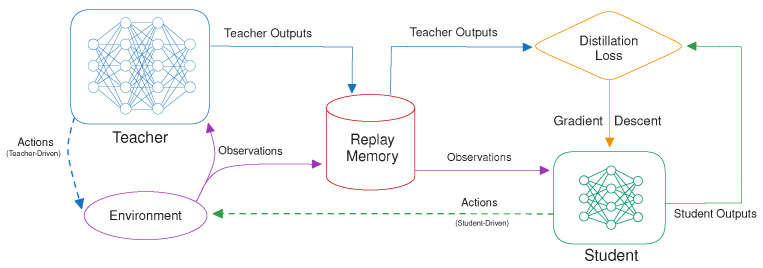
An illustration of the policy distillation algorithm.

In this original definition of the policy distillation [12] loss for DQN teachers, the network outputs (*q*) correspond to a list of Q-values, one for each possible action. The softmax function is used to transform these Q-values into a probability vector over the actions, to be used as input for the KL-divergence. This results in Algorithm 1 and Figure 3.

Other work has extended this approach for use in combination with actor-critic teacher algorithms for discrete action spaces by applying a similar KL-divergence loss between the two policies directly [15] and optionally including an additional term to learn the critic values [14]. In this paper, we look at how to adapt this loss for the distillation of continuous actions.
**Algorithm 1:** Policy Distillation
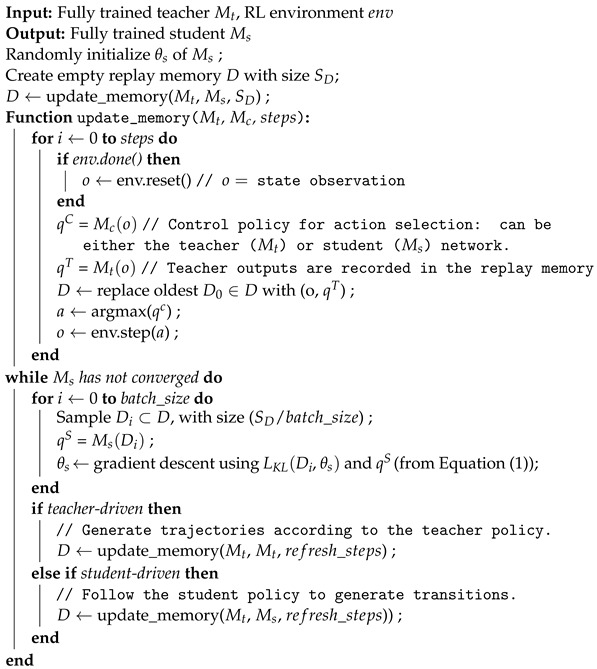


## 3. Related Work

Several existing papers have already employed some form of model distillation in combination with continuous action spaces, but most of these methods do not learn the teacher policy directly, so they would not strictly be classified as policy distillation. Instead, the state-value function that is also learned by actor-critic teachers is used for bootstrapping, replacing the student’s critic during policy updates. This has also been described by Czarnecki et al. [13] for discrete action spaces, but they note that this method saturates early on for teachers with suboptimal critics. Xu et al. [23] take this approach for a multi-task policy distillation, where a single agent is trained based on several teachers that are each specialized in a single task, to train a single student that can perform all tasks. They first used an MSE loss to distill the critic values of a TD3 teacher into a student with two critic heads. These distilled values are later used to train the student’s policy instead of using the teacher’s critic directly as proposed by Czarnecki et al. [13]. Lai et al. [24] propose a similar method but in a setting that would not typically be classified as distillation, with two students and no teacher. These two students learn independently based on a traditional actor-critic RL objective but use the peer’s state-value function to update their actor instead of using their own critic if the peer’s prediction is more advantageous for a given state.

Our work differs from these methods by learning from the actual policy of the teacher instead of indirectly from the value function. This more closely maintains student fidelity to their teacher [25] and allows us to more effectively distill and maintain a stochastic policy. The state-value function predicts the expected (discounted) return when starting in a certain state and following the associated policy [26]. This provides an estimate of how good it is to be in a certain state of the environment, which is used as a signal to update the policy (or actor) towards states that are more valuable. It is not intrinsically aware of the concept of actions, however, so it cannot model any behavior indicating which actions are viable in a given state. The student therefore still needs to learn their own policy under the guidance of the teacher’s critic using a traditional DRL algorithm, preferably the same that was used to train the teacher. Often, the critic requires more network capacity than the actor, so using the larger critic from the teacher instead of the student’s own critic could be beneficial for learning [27]. However, the critic is no longer necessary during inference when the student is deployed and can therefore be removed from the architecture to save resources, eliminating any potential improvement in network size. The general concept of distillation for model compression, where the knowledge of a larger model is distilled into a smaller one, does not apply here. Instead, these existing works focus on different use cases, such as multi-task or peer learning, where this approach is more logical. We therefore focus on distilling the actual learned behavior of the teacher in the form of the policy, as our goal is compression for low-power inference on edge devices.

Berseth et al. [28] also distill the teacher policy directly in their PLAID method, but by using an MSE loss to only transfer the mean action, any policy is reduced to being deterministic. Likewise, their method is designed for a multi-task setting, without including any compression. We included this method as a baseline in our experiments and proposed a similar function based on the Huber loss for teachers that perform best when evaluated deterministically, but our focus is on the distillation of stochastic policies. Learning this stochastic student policy also has an impact on the distribution of transitions collected in the replay memory when a student-driven control policy is used, so we compare this effect to the traditional teacher-driven method.

## 4. Methodology

**Figure 4:**
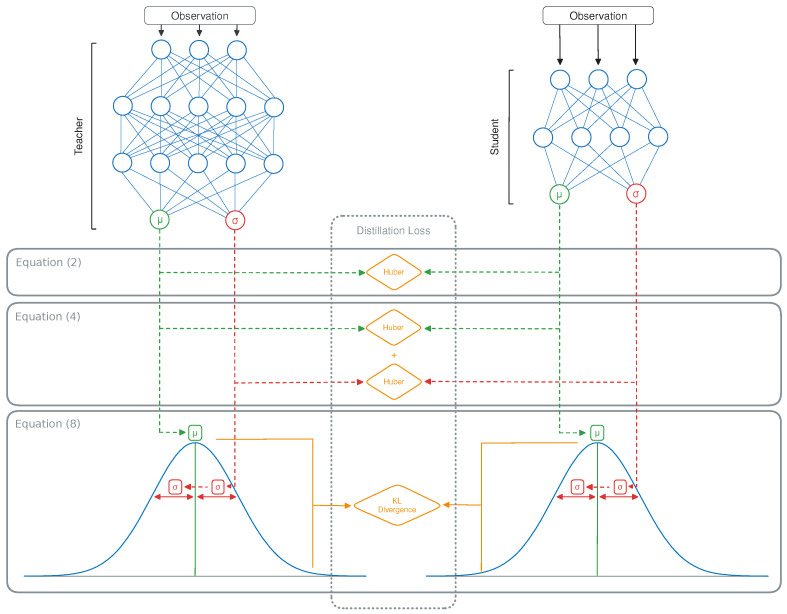
An illustration of the proposed distillation losses.

We propose several loss functions for the distillation of continuous actions based on a combination of the teacher’s mean (μ) and standard deviation (σ), with an overview given in Figure 4. Such a loss function should accurately define the similarity between the two policies, based on the action distributions predicted by both networks, with a lower value corresponding to the student matching their teacher’s behavior more closely. The expected effectiveness of the proposed losses depends on whether the teacher performs best in a deterministic or stochastic evaluation and whether a teacher-driven or student-driven setting is used.

### 4.1. Distilling the Mean Action

The first loss is the simplest, serving as a baseline that is mostly useful in combination with deterministic teachers in the teacher-driven scenario or in case σ was not learned by the model. It consists of only distilling the mean action that the student needs to follow, resulting in a fully deterministic policy, similar to what is proposed by Berseth et al. [28]. The original policy distillation loss (Equation (1)) is based on the KL-divergence between two probability vectors, which cannot be used for learning a single mean value for each action [12]. Instead, we propose to use the Huber loss between the mean of the student ($\mu^{S}$) and the teacher ($\mu^{T}$) for each action (*a*):

(2) $$L\left(D,\theta_{S}\right)=\sum_{i=1}^{\left|D\right|}\sum_{a\in A}\mathrm{Huber}\left(\mu_{i,a}^{S},\mu_{i,a}^{T},1\right)$$

(3) $$Huber\left(a,b,\delta \right)=\left\{\begin{array}{cc} \frac{1}{2}{\left(a-b\right)}^{2} & \mathrm{for}\;|a-b|\leq \delta ,\\ \delta \cdot \left(|a-b|-\frac{1}{2}\delta \right), & \mathrm{otherwise}. \end{array}\right.$$

We chose to make use of the Huber loss for this baseline instead of the MSE loss used by Berseth et al. [28] since it is less sensitive to outliers and has a smoother slope for larger values, resulting in it outperforming the MSE loss in our initial experiments.

### 4.2. Distilling the Mean Action and Its Standard Deviation

Some teachers perform better when actions are sampled stochastically, in which case the student should also learn the value of σ to perform optimally. By learning when precise action is required and when actions can be taken more stochastically, the agent can also build a deeper understanding of its environment. This could be seen as a different form of ‘dark’ knowledge, similar to the distribution of alternative actions in policy distillation for discrete action spaces [12]. Learning σ is even more important when using student-driven policy distillation, as this allows the student to explore more of the state space to learn multiple viable ways to obtain a high return, further enhancing its representation of the task dynamics. In effect, it enables the exploration–exploitation trade-off to apply in a distillation context, where the mean action would focus purely on exploitation. This has the potential to increase generalization and robustness against changes in the environment. In turn, this enables the student to recover more gracefully from mistakes caused by the remaining distribution shift, leading to increased task performance, as reflected by a higher return. It can also help prevent the student from becoming stuck in local minima, where the teacher is less knowledgeable and provides inaccurate behavior. By encouraging the student to deviate more from this suboptimal strategy, it can move towards a region in the state space where the teacher’s guidance is more effective.

If σ is state-independent, this vector can simply be copied from the teacher to the student while continuing to train using Equation (2). Note that in this case, it would likely be beneficial to train the teacher using a state-dependent standard deviation instead. Otherwise, we include an additional term for distilling σ, with λ a scaling factor to ensure that the loss for σ does not dominate over the one for μ or the other way around:

(4) $$L\left(D,\theta_{S}\right)=\sum_{i=1}^{\left|D\right|}\sum_{a\in A}\mathrm{Huber}\left(\mu_{i,a}^{S},\mu_{i,a}^{T},1\right)+\lambda \cdot \mathrm{Huber}\left(\sigma_{i,a}^{S},\sigma_{i,a}^{T},1\right)$$

Since we have access to both μ and σ, these can be used to once again define a probability distribution. This also allows the student to sample actions based on N(μ, σ), which was not possible when trained using Equation (2). The Huber loss is still not optimally suited for defining a distance metric between two probability distributions however; it simply defines a distance between the two values separately, without any context of how they are used together.

### 4.3. Distilling the Action Distribution

In traditional policy distillation, the student is also trained using a probability distribution over actions, where this is defined using the probability vector given by the teacher outputs [12]. Instead of learning to reproduce the same precise values as the teacher, such as what we proposed in Equation (2) for learning the mean action, the student outputs are shaped to produce a similar probability curve. This is achieved through a derivation of the KL-divergence for discrete probability distributions that is most often used in the context of deep learning. In the context of continuous actions, the network outputs are not in the form of probability vectors, so this loss cannot be applied to this setting. Instead, we derive the KL-divergence between two absolutely continuous univariate normal distributions, starting with the general definition of the KL-divergence for distributions P and Q of continuous random variables:

(5) $$\mathrm{KL}\left(P,Q\right)=\int p\left(x\right)\log\left(\frac{p(x)}{q(x)}\right)dx$$

In our setting, P and Q are normal distributions defined by μ and σ, for which the probability density function is defined as follows:

(6) $$\begin{array}{cc} f(x) & =\frac{1}{\sigma \sqrt{2\pi }}e^{-\frac{1}{2}{\left(\frac{x-\mu }{\sigma }\right)}^{2}} \end{array}$$

To substitute this in Equation (5), we first focus on the log division:

$$\begin{array}{cc} \log\left(\frac{p(x)}{q(x)}\right) & =\log p(x)-\log q(x)\\ \; & =\left[\log\left(\frac{1}{\sigma_{p}\sqrt{2\pi }}e^{-\frac{1}{2}{\left(\frac{x-\mu_{p}}{\sigma_{p}}\right)}^{2}}\right)-\log\left(\frac{1}{\sigma_{q}\sqrt{2\pi }}e^{-\frac{1}{2}{\left(\frac{x-\mu_{q}}{\sigma_{q}}\right)}^{2}}\right)\right]\\ \; & =\left[-\frac{1}{2}\log\left(2\pi \right)-\log\left(\sigma_{p}\right)-\frac{1}{2}{\left(\frac{x-\mu_{p}}{\sigma_{p}}\right)}^{2}+\frac{1}{2}\log\left(2\pi \right)+\log\left(\sigma_{q}\right)+\frac{1}{2}{\left(\frac{x-\mu_{q}}{\sigma_{q}}\right)}^{2}\right]\\ \; & =\left[\log\left(\frac{\sigma_{q}}{\sigma_{p}}\right)+\frac{1}{2}\left[{\left(\frac{x-\mu_{q}}{\sigma_{q}}\right)}^{2}-{\left(\frac{x-\mu_{p}}{\sigma_{p}}\right)}^{2}\right]\right] \end{array}$$

The full equation then becomes:

(7) $$\mathrm{KL}\left(P,Q\right)=\int \frac{1}{\sigma_{p}\sqrt{2\pi }}e^{-\frac{1}{2}{\left(\frac{x-\mu_{p}}{\sigma_{p}}\right)}^{2}}\left[\log\left(\frac{\sigma_{q}}{\sigma_{p}}\right)+\frac{1}{2}\left[{\left(\frac{x-\mu_{q}}{\sigma_{q}}\right)}^{2}-{\left(\frac{x-\mu_{p}}{\sigma_{p}}\right)}^{2}\right]\right]dx$$

We then rewrite this using the expectation with respect to distribution P:

$$\begin{array}{cc} \mathrm{KL}(P,Q) & =E_{p}\left[\log\left(\frac{\sigma_{q}}{\sigma_{p}}\right)+\frac{1}{2}\left[{\left(\frac{x-\mu_{q}}{\sigma_{q}}\right)}^{2}-{\left(\frac{x-\mu_{p}}{\sigma_{p}}\right)}^{2}\right]\right]\\ \; & =\log\left(\frac{\sigma_{q}}{\sigma_{p}}\right)+\frac{1}{2\sigma_{q}^{2}}E_{p}\left[{\left(X-\mu_{q}\right)}^{2}\right]-\frac{1}{2\sigma_{p}^{2}}E_{p}\left[{\left(X-\mu_{p}\right)}^{2}\right]\\ \; & =\log\left(\frac{\sigma_{q}}{\sigma_{p}}\right)+\frac{1}{2\sigma_{q}^{2}}E_{p}\left[{\left(X-\mu_{q}\right)}^{2}\right]-\frac{1}{2} \end{array}$$

Note that we could rewrite ${\left(X-\mu_{q}\right)}^{2}$ to:

$$\begin{array}{cc} {\left(X-\mu_{q}\right)}^{2} & ={\left(X-\mu_{p}+\mu_{p}-\mu_{q}\right)}^{2}\\ \; & ={\left(\left(X-\mu_{p}\right)+\left(\mu_{p}-\mu_{q}\right)\right)}^{2}\\ \; & ={\left(X-\mu_{p}\right)}^{2}+2\left(\mu_{p}-\mu_{q}\right)\left(X-\mu_{p}\right)+{\left(\mu_{p}-\mu_{q}\right)}^{2} \end{array}$$

Substituting this results in:

$$\begin{array}{cc} \mathrm{KL}(P,Q) & =\log\left(\frac{\sigma_{q}}{\sigma_{p}}\right)+\frac{1}{2\sigma_{q}^{2}}E_{p}\left[{\left(X-\mu_{p}\right)}^{2}+2\left(\mu_{p}-\mu_{q}\right)\left(X-\mu_{p}\right)+{\left(\mu_{p}-\mu_{q}\right)}^{2}\right]-\frac{1}{2}\\ \; & =\log\left(\frac{\sigma_{q}}{\sigma_{p}}\right)+\frac{1}{2\sigma_{q}^{2}}\left(E_{p}\left[{\left(X-\mu_{p}\right)}^{2}\right]+2\left(\mu_{p}-\mu_{q}\right)E_{p}\left[(X-\mu_{p})\right]+{\left(\mu_{p}-\mu_{q}\right)}^{2}\right)-\frac{1}{2}\\ \; & =\log\left(\frac{\sigma_{q}}{\sigma_{p}}\right)+\frac{\sigma_{p}^{2}+{\left(\mu_{p}-\mu_{q}\right)}^{2}}{2\sigma_{q}^{2}}-\frac{1}{2} \end{array}$$

Finally, we apply this for policy distillation of continuous action spaces:

(8) $$L_{KL}\left(D,\theta_{S}\right)=\sum_{i=1}^{\left|D\right|}\sum_{a\in A}\log\frac{\sigma_{i,a}^{T}}{\sigma_{i,a}^{S}}+\frac{{\left(\sigma_{i,j}^{S}\right)}^{2}+{\left(\mu_{i,a}^{S}-\mu_{i,a}^{T}\right)}^{2}}{2{\left(\sigma_{i,a}^{T}\right)}^{2}}-\frac{1}{2}$$

This should allow for a smoother optimization objective than learning both values using the Huber loss, similar to the distillation of discrete actions.

## 5. Experimental Setup

### 5.1. Evaluation Environments

**Figure 5:**
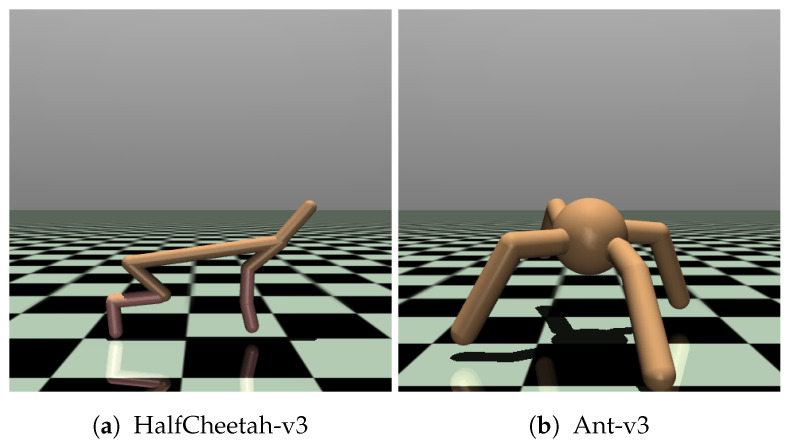
A graphical render of the environments used in this paper.

We evaluate the effectiveness of the loss functions proposed in Section 4 in two continuous control environments (Ant-v3 and HalfCheetah-v3) that are part of the Gymnasium project [29], shown in Figure 5. These environments were chosen as they are arguably the two most prevalent benchmarks in the MuJoCo suite, the de facto standard for continuous control tasks in DRL research. This allowed us to apply our methods to compress publicly available state-of-the-art DRL models, making it straightforward to compare to existing work and strongly increasing reproducibility.

Environments in this suite have complex, high-dimensional continuous state and action spaces and require sophisticated control strategies [29]. This takes the form of a physics simulation of a robot with a specific morphology and complex dynamic interaction between multiple joints that need to be efficiently coordinated. Distilling a policy that consists of multiple coordinated continuous actions allows us to verify that our proposed loss functions are robust to even the most complex policy architectures. The relationship between actions (torques) and the resulting state of the creature (positions, velocities, angles) is non-linear, making it challenging to learn a compact policy that captures these complex dynamics effectively. The difficulty of these tasks should result in a pronounced difference in the average return obtained by students of different sizes, allowing us to compare the impact of the compression level for each of the proposed methods. By relying on a physics engine that accurately models advanced dynamics [30], these environments are representative of many real-world continuous control tasks, such as robotics and drone control that require low-power operation at the edge.

The goal in the two chosen environments is to achieve stable locomotion of the robot by applying torques to its joints to move through the environment as quickly as possible. In HalfCheetah-v3, this robot takes the form of a 2D bipedal creature with six controllable joints and therefore six separate continuous values in the action space that need to be distilled. The observation space consists of 17 continuous variables, including the positions and velocities of its limbs and the angles of its joints. Being inspired by a cheetah, the goal of the agent is to run as fast as possible, even if this means sacrificing stability. The movement of the 3D quadrupedal creature with eight controllable joints in Ant-v3 needs to be more sophisticated to achieve a high return. In addition to being rewarded for efficient forward movement, it needs to balance itself by keeping its torso within a certain height range. If it falls over, the episode is terminated early. It also has a higher-dimensional observation space of 111 continuous variables that now also includes the contact forces applied to the center of mass of each of the body parts. Combined, these tasks require an effective balance between exploration and exploitation to learn how to optimally coordinate the different joints to move quickly while maintaining stability to not fall over. A stochastic policy can be beneficial for both these aspects by exploring the state space more effectively and increasing the robustness to recover from unstable configurations.

### 5.2. Model Architectures

To use as teachers for training our students and as baselines for uncompressed models, we make use of two state-of-the-art DRL algorithms: SAC [11] and PPO. For increased reproducibility, we make use of publicly available pre-trained agents from the Stable Baselines3 project [31] for our teacher networks. These teachers have different behaviors when evaluated either deterministically or stochastically. Although both are trained to learn a stochastic policy, a PPO agent often performs better during final evaluation when the mean action is chosen, whereas an SAC agent performs better when actions are sampled stochastically. This can be seen in Table 1, which shows the average return and standard deviation for 200 episodes using our teacher models on the used environments and for both evaluation methods.

The Stable Baselines3 project uses a different network size for these teachers depending on the environment, as shown in Table 2. Note that we did not include the critic head for the teacher sizes in this table, as this is not required for inference. It also shows the parameter count for one of our student architectures (with ID 6) in all its configurations. This student was used to compare our proposed loss functions and the chosen control policy, as this was the smallest architecture where the number of parameters was not yet a limiting factor.

The impact of the compression level is further evaluated using ten different student architectures, for which the number of layers, neurons per layer, and total number of parameters are shown in Table 3. This ranges from 0.67% to 189% of the size of the SAC teacher. In case a loss function is used that includes the standard deviation, the last layer will have two heads: one for the mean actions and one for the standard deviations.

### 5.3. Training Procedure

Each experiment runs for 200 training epochs, with 1 epoch being completed when the student has been updated based on all 100,000 transitions in the replay memory *D*. Transitions are sampled in random order from *D* in sets of mini-batches with size 64. After each epoch, the student is evaluated for 50 episodes while collecting the average return, with the action selection being stochastic or deterministic, based on which performs best for this teacher model (see Table 1). The oldest 10% of the transitions in *D* are then also replaced by new environment interactions after each epoch. These environment interactions are either student-driven or teacher-driven, based on the current configuration of the experiment. Each experiment configuration is repeated for five independent runs, resulting in the mean and corresponding standard deviations of the return and entropy at each epoch discussed in Section 6.

We compute the entropy of the action distribution predicted by our students during testing to more tangibly evaluate how well the stochasticity of the teacher is maintained as part of the distillation process. Since these are continuous univariate normal distributions, we compute this using:

(9) $$H\left(x\right)=\frac{1}{2}\log\left(2\pi \sigma^{2}\right)+\frac{1}{2}$$

For a random variable x∼N(μ,σ), i.e., the action prediction. This entropy is then averaged over all steps in a trajectory.

## 6. Results and Discussion

In this section, we investigate the effectiveness of the three loss functions proposed in Section 4 under various circumstances. We start by performing an ablation study to isolate the effects of the chosen loss function, the control policy, and finally the teacher algorithm. This will provide us with a better understanding of how each of these components in our methodology impacts the training process and how they interact with each other to culminate in the final policy behavior. This is measured in terms of the average return, but we also analyze the entropy of the action distribution to evaluate how well the stochasticity of the teacher is maintained as part of the distillation process. Afterward, we perform a sensitivity study of our methodology for different compression levels to evaluate the impact of the student size on the final policy performance. Finally, we analyze the runtime performance in terms of inference speed of each of the student architectures to gain a better understanding of the trade-offs between the different student sizes.

### 6.1. Distillation Loss

To isolate the impact of the chosen distillation loss, we compare the average return of students trained using each of the three proposed loss functions, with the same SAC teacher and a student-driven control policy. Distilling a stochastic policy (learning σ) and using this to collect training data will increase exploration and therefore widen the state distribution in the replay memory. If the student is trained using Equation (2) instead, the replay memory will only contain deterministic trajectories, which are not always optimal (see Table 1).

**Figure 6:**
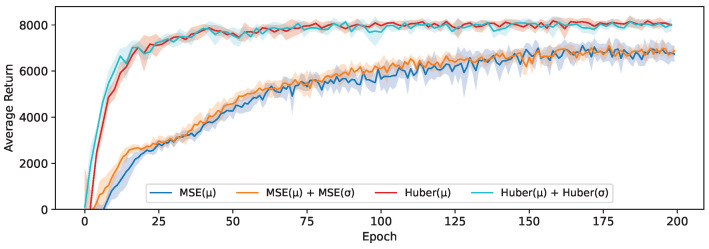
The average return obtained using either an MSE or Huber-based distillation loss.

As a baseline, we start by comparing the MSE-based loss originally proposed by Berseth et al. [28] between mean actions μ to our Huber-based loss functions ((2) and (4)), as well as an analogous MSE loss with both μ and σ. The results of this are shown in Figure 6. The students trained using our baseline Huber-based loss converge much more quickly and obtain an average return that is 18% higher on average. This confirms the benefit in the context of distillation of the Huber loss being less sensitive to outliers and having a smoother slope for larger values. However, it is also notable that learning the state-dependent value of σ through an auxiliary MSE or Huber loss does not yield any noticeable benefit; instead, this results in a comparable average return to when only the mean action is distilled in this experiment.

**Figure 7:**
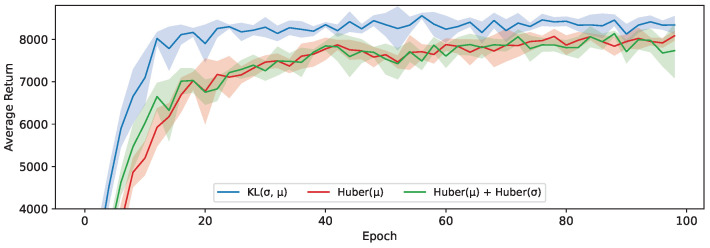
The average return of 5 students trained using student-driven distillation on the HalfCheetah-v3 environment with an SAC teacher.

Looking at Figure 7, we do see that our proposed loss based on the KL-divergence (Equation (8)) to transfer the action distribution performs significantly better than those based on a Huber loss. This does align with our hypothesis that the loss landscape when shaping the probability distribution of the student to match that of the teacher more closely is smoother than learning the two concrete values independently, leading to a better optimization. Learning these values separately, as was the case in Figure 6, precisely enough to accurately model the distribution might also require more capacity, resulting in this approach suffering more heavily from the limited capacity of the student. We test this conclusion more extensively in Section 6.4 by comparing these results with different student sizes.

### 6.2. Control Policy

Using a student-driven control policy will result in a different distribution of transitions in the replay memory compared to when using a teacher-driven control policy, where the distribution shift between the training and testing data is more pronounced. In the student-driven setting, the initial distribution will be less accurate and more exploratory but will gradually converge to the teacher distribution as the student learns. To test this hypothesis for continuous actions, we ran the same experiment as in the previous section, but this time with a teacher-driven control policy.

#### 6.2.1. Impact on Average Return

**Figure 8:**
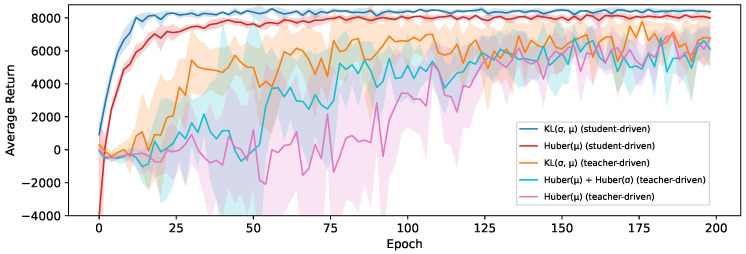
The average return during training for student and teacher-driven distillation on the HalfCheetah-v3 environment and an SAC teacher.

The effects of this distribution shift can be seen in Figure 8, which shows the average return for both control policies and all loss functions on the HalfCheetah-v3 environment. The experiments with a teacher-driven action selection perform significantly worse than their student-driven counterparts. This also results in far more variance in performance between epochs, and it takes much longer to converge. As the distillation loss becomes smaller, the students will behave more similarly to their teacher and the distribution shift will eventually reduce, but never disappear completely. Eventually, the students in the teacher-driven configuration converge on a similar obtained average return, regardless of the used loss function. However, there is a clear order in how quickly the students reach this convergence point, with the agents trained using our loss based on the KL-divergence (Equation (8)) being considerably more sample efficient, followed by the agent trained using the Huber loss for both μ and σ (Equation (4)) and finally the agent that only learns a deterministic policy in the form of the mean actions μ (Equation (2)). This suggests that even though learning a stochastic policy is beneficial in this setting, the remaining distribution shift eventually becomes the limiting factor that causes all students to hit the same performance ceiling. We find that for this environment, the difference between the used control policy is more pronounced than the difference between the used loss functions but that the KL-divergence loss is still the most effective choice.

**Figure 9:**
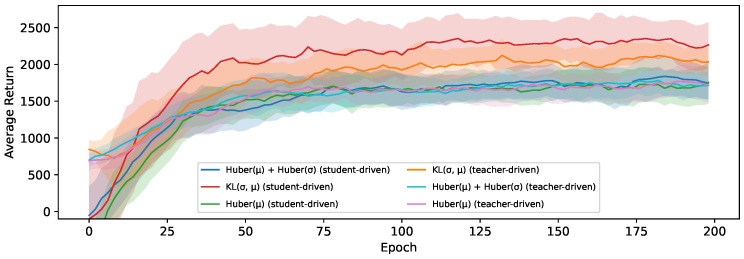
The exponential running mean of the average return during training for student and teacher-driven distillation on the Ant-v3 environment and an SAC teacher.

Figure 9 also shows the average return for all configurations, but this time on the Ant-v3 environment instead. The distribution shift is less pronounced in this environment, resulting in the gap between student and teacher-driven action selection disappearing for all but the students trained using our KL-divergence distillation loss, where using a student-driven control policy still has a noticeable benefit. These are also the two configurations that stand out from the others, with a significantly higher return on average, confirming the same conclusion as on the HalfCheetah-v3 environment that this loss function is the best out of the three considered options for the distillation of continuous actions with a stochastic teacher. Note that the variance of the average return between epochs is much higher for this environment, so we show the exponential running mean with a window size of 10 in these plots to gain a clearer impression of the overall performance when using each of the loss functions.

#### 6.2.2. Impact on Policy Entropy

**Figure 10:**
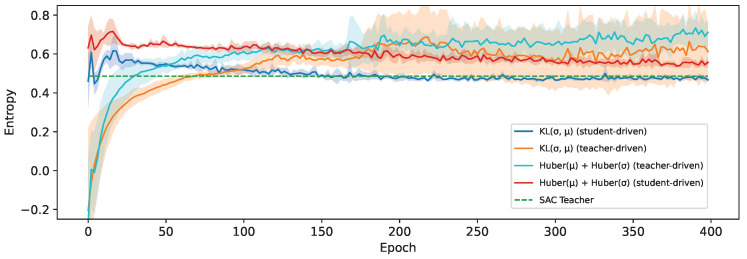
The average entropy measured for students trained using a loss that includes σ on the HalfCheetah-v3 environment and an SAC teacher.

We hypothesize that this difference in performance between the distillation losses is mainly due to the maintained accuracy of the policy stochasticity. This has particular importance to reach a high degree of fidelity with teachers such as SAC, which are optimized to maximize an entropy-regularized return [11]. To verify this, we measure the entropy of the action distribution predicted by the students during testing, as can be seen in Figure 10. This clearly shows that the relative order of the experiments is the same as for the average return, but in reverse. The student trained using our KL-divergence-based distillation loss indeed matches the entropy of the teacher the closest, and the more similar the entropy is to the teacher, the higher the average return that is obtained. However, it seems that the entropy is overestimated when using the other losses, resulting in more actions being taken that deviate too much from the teacher policy.

So, the KL-divergence loss strikes a good balance between learning a stochastic policy, which our results confirm is optimal for this teacher, while staying closer to the teacher policy by not overestimating the entropy either. In the student-driven experiments, the entropy is initially higher, before gradually converging to the teacher entropy. This is beneficial for training, as the data collected during the first epochs will contain more exploratory behavior and thus results in faster learning and reducing once the control policy is stabilizing. These values are also a lot more stable and have much less inter-run variance compared to the teacher-driven experiments, which only seem to become worse over time.

### 6.3. Teacher Algorithm

In this section, we evaluate how generic our proposed methods can be applied with different teacher algorithms, focusing on the two most commonly used for continuous control tasks: SAC and PPO. The SAC algorithm tries to optimize a policy that obtains the highest return while staying as stochastic as possible. With PPO, on the other hand, the entropy generally decreases over time, as it converges on a more stable policy. This translates into the PPO teacher achieving a higher average return when evaluated deterministically, while the SAC teacher performs better when actions are sampled stochastically, as shown earlier in Table 1. We have demonstrated in the previous sections that the KL-divergence loss is the most effective for distilling a stochastic policy, but it remains to be seen if this benefit remains for more deterministic teachers.

**Figure 11:**
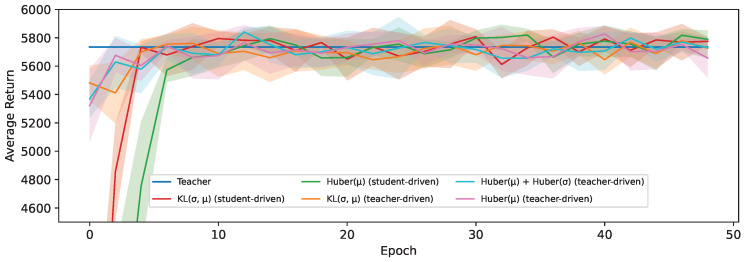
The average return during training for student and teacher-driven distillation on the HalfCheetah-v3 environment and a PPO teacher.

**Figure 12:**
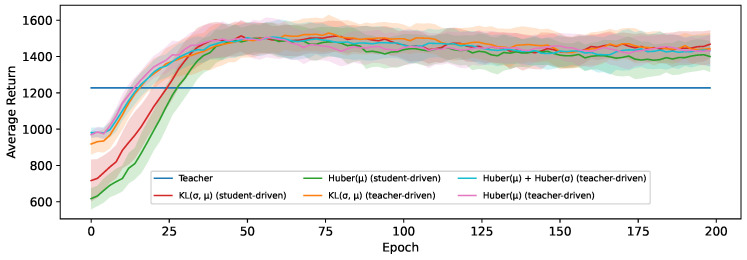
The exponential running mean of the average return during training for student and teacher-driven distillation on the Ant-v3 environment and a PPO teacher.

Therefore, we present the distillation results with a PPO teacher in Figure 11 on the HalfCheetah-v3 environment and in Figure 12 on the Ant-v3 environment. This shows virtually no difference in the used loss functions or the used control policy for action selection. Since the PPO teacher performs best when evaluated deterministically, there appears to be no benefit in learning the state-dependent value of σ if it is no longer used at evaluation time. By following a deterministic policy, the student is also less likely to end up in an unseen part of the environment, thereby reducing the difference between a student-driven and teacher-driven setting.

What is more notable about the PPO results however is that the students outperform their teacher on the Ant-v3 environment. In the context of policy distillation for discrete action spaces, this phenomenon has also been observed and attributed to the regularization effect of distillation [12]. These students (Figure 12) reach a peak average return after being trained for around 37 epochs, but this slowly starts to decline afterward, while their loss continues to improve. A lower loss generally indicates that the students behave more similarly to their teacher, which in this case is detrimental, resulting in regression.

This outcome relates to the work by Stanton et al. [25], who have shown in a supervised learning context that knowledge distillation does not typically work as commonly understood where the student learns to exactly match the teacher’s behavior. There is a large discrepancy in the predictive distributions of teachers and their final students, even if the student has the same capacity as the teacher and therefore should be able to match it precisely. During these experiments, the generalization of our students first improves, but as training progresses, this shifts to improving their fidelity.

The students that were trained based on an SAC teacher performed slightly worse compared to their teacher on the HalfCheetah-v3 environment, and a more significant performance hit was observed on the Ant-v3 environment. This is likely due to the level of compression being significantly higher compared to the PPO distillation for this environment, as the student architecture is kept constant in this section to isolate the impact of the loss function choice.

### 6.4. Compression Level

**Figure 13:**
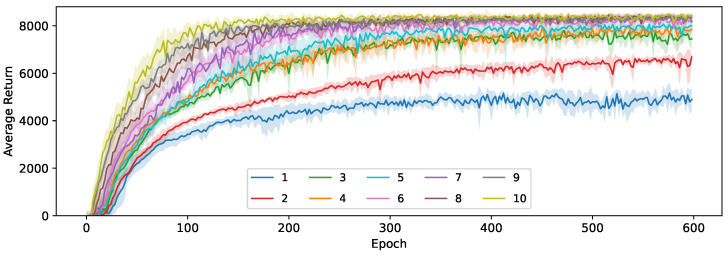
The average return for 10 student sizes during training using Equation (8) (KL).

**Figure 14:**
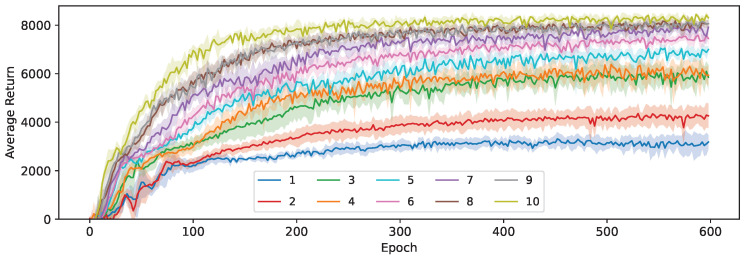
Average return for 10 student sizes during training using Equation (4) (Huber).

We investigate the compression potential of our methods by repeating the experiments in Section 6.1 for a wide range of student network sizes, as listed in Table 3. In Figure 13, our loss based on the KL-divergence (Equation (8)) was used, while Figure 14 shows the results when using the Huber-based loss for both μ and σ. Using our KL-based loss, we can reach a compression of 7.2× (student 6) before any noticeable performance hit occurs. The average return stays relatively high at up to 36.2× compression (student 3), before dropping more significantly at even higher levels of compression. When going from student 3 to student 2, we also reduce the number of layers in the architecture from 3 to 2, which becomes insufficient to accurately model the policy for this task. The convergence rate noticeably decreases at each size step, with student 2 still improving even after 600 epochs.

The impact of the student size is much higher when using the Huber-based distillation loss. There is still a noticeable difference between the average return obtained by the largest (10) and second largest (9) student, even though this largest student is actually 2× larger than their teacher for this environment. This makes this loss particularly unsuited for distillation, as it requires more capacity than the original SAC teacher algorithm to reach the highest potential average return. The largest student (10) here still performs slightly worse than the fourth-largest student (7) when trained based on the KL-divergence loss, but the performance gap does almost disappear for networks that approach the teacher in size. This means that our Huber-based distillation loss can still effectively transfer the teacher’s knowledge to the student, but it requires considerably more capacity to learn two values (μ and σ) independently, making it infeasible for compression purposes. The convergence rate of these students is also slower, making it more computationally expensive at training time.

Therefore, we conclude that both proposed loss functions can be effective at distilling the stochastic continuous behavior of the teacher, but the efficiency in terms of required network size and number of samples is significantly higher for our loss based on the KL-divergence, to the extent that the Huber-based loss becomes impractical for compression.

### 6.5. Runtime Performance

Finally, we analyze how this compression to the various student architectures (see Table 3) translates to benefits in terms of real-world performance. Note that we focus on the inference performance of the final student models, as the training procedure is not intended to run on these low-power devices. This is measured on a range of low and high-power devices by sequentially passing a single observation 10,000 times through the network, which is then repeated 10 times using a random order of network sizes to ensure that any slowdown due to the prolonged experiment does not bias the results of a particular size. We then report the average number of steps per second, as shown in Table 4. Note that student 9 uses the same architecture as the SAC teacher, and student 10 is similar in size to the PPO teacher, so these are used as a baseline.

An important observation is that although the model performance in terms of average return scales with the number of parameters in the model, the story is more complicated when looking at the runtime performance. Notably, student 7 is the slowest network for most devices, even though it is only 13% as big as the largest network. It does however have the most network layers, being six compared to only four for student 10. This was chosen to keep a consistent increase of about 2× parameters when going from one size to the next while keeping the number of neurons per layer as a power of 2. A similar result can be seen for student 4, which also has one more layer than the surrounding ones. Having a deeper network limits the potential for parallelization on devices with many computational units, such as GPUs or multi-core CPUs, while we did not notice a clear benefit of using more than three layers on the average return. On the lowest-power device we tested (Raspberry Pi), this difference due to the number of layers is less pronounced and the total network size becomes more important.

For high-power devices or ones designed for many parallel operations, the effective speed gain obtained by compressing these models is relatively minor, improving by only 9% worst case for a reduction to a mere 0.6% of the original size. In these cases, the overhead involved in simply running a model at all becomes the bottleneck, independent of the model itself up to a certain size. The highest improvement can therefore also be seen on the lowest-power device, the Raspberry Pi 3B, in which we can see a maximal runtime improvement of 64% compared to the SAC teacher or 109% compared to the PPO one. At this size, however, the model is no longer able to solve the task nearly as well as the teacher, so a comparison to student 3 with a runtime improvement of 44% and 85%, respectively, is more reasonable.

It is also worth noting that there is more to runtime performance, for which you might want to apply model compression than purely the achieved number of steps per second. Often, when running on embedded devices, there are additional constraints in terms of memory or power consumption, or on devices with hardware acceleration for neural network inference there can be a limit to the number of supported layers or parameters. In this setting, model compression can enable the use of more advanced models on devices that would otherwise not be capable of running them due to memory constraints. There, the model size in bytes becomes an important metric that impacts portability rather than performance. This can simply be derived for our models by taking the parameter count reported in Table 3 and multiplying it by 4. The popular Arduino Uno R3 microcontroller, for example, has only 32 kB of available ROM [32], which is only enough to store up to student 5, with a size of 24 kB.

Measuring the direct impact of policy distillation on power consumption improvements is less straightforward, as this is more a property of the hardware than the individual model. You can force the device to periodically switch to a lower power state by artificially limiting the frame rate, but this difference is usually negligible compared to a switch in hardware class [33]. Instead, to optimize for this, we suggest searching for the hardware with the lowest power consumption that can still run the compressed model at an acceptable speed. For example, with a target of 600 steps per second, the Raspberry Pi 3B consumes around 4.2 W [33] and student 3 is a valid option. It will consume the same power when running the original model but at half the inference speed. If the target is 800 steps per second, however, a jump to an Nvidia Jetson TX2 running at 15 W [34] becomes necessary.

We conclude this section by arguing the importance of carefully designing the architecture of the model with your target device in mind, performing benchmarks to evaluate the best option that meets your runtime requirements, and applying our proposed distillation method based on the KL-divergence to achieve the best model for your use case. Optionally, a trade-off can be made between the average return and steps per second to achieve the best result.

## 7. Conclusions

Deploying intelligent agents for continuous control tasks, such as drones, AMRs, or IoT devices, directly on low-power edge devices is a difficult challenge, as their computational resources are limited, and the available battery power is scarce. This paper addressed this challenge by proposing a novel approach for compressing such DRL agents by extending policy distillation to support the distillation of stochastic teachers that operate on continuous action spaces, whereas existing work was limited to deterministic policies or discrete actions. Not only does this compression increase their applicability while reducing associated deployment costs, but processing the data locally eliminates the latency, reliability, and privacy issues that come with wireless communication to cloud-based solutions.

To this end, we proposed three new loss functions that define a distance between the distributions from which actions are sampled in teacher and student networks. In particular, we focused on maintaining the stochasticity of the teacher policy by transferring both the predicted mean action and state-dependent standard deviation. This was compared to a baseline method where we only distill the mean action, resulting in a completely deterministic policy. We also investigated how this affects the collection of transitions on which our student is trained by evaluating our methods using both a student-driven and teacher-driven control policy. Finally, the compression potential of each method was evaluated by comparing the average return obtained by students of ten different sizes, ranging from 0.6% to 189% of their teacher’s size. We then showed how each of these compression levels translates into improvements in real-world run-time performance.

Our results demonstrate that especially our loss based on the KL-divergence between the univariate normal distributions defined by μ and σ is highly effective at transferring the action distribution from the teacher to the student. When distilling an SAC teacher, it outperformed our baseline where only the mean action is distilled on average by 8% on the HalfCheetah-v3 environment and 34% on Ant-v3. This effect is especially noticeable in the student-driven setting, but we were also able to observe a significant increase in sample efficiency in the teacher-driven setup. When a less stochastic PPO teacher was used, all our proposed methods performed equally well, managing to maintain or even outperform their teacher while being significantly smaller. This also confirms that the regularization effect of policy distillation that was observed in the setting for discrete action spaces still holds for the continuous case.

In general, we recommend a student-driven distillation approach with our loss based on the KL-divergence between continuous actions as the most effective and stable compression method for future applied work. Through this method, DRL agents designed to solve continuous control tasks were able to be heavily compressed by up to 750% without a significant penalty to their effectiveness.

## Figures and Tables

**Table 1:** Average return and standard deviation for our PPO and SAC teachers on the chosen environments using either stochastic or deterministic action selection.

Network	Ant-v3	HalfCheetah-v3
SAC Stochastic	4682 ± 1218	9010 ± 113
SAC Deterministic	1797 ± 993	8494 ± 186
PPO Deterministic	1227 ± 483	5735 ± 723
PPO Stochastic	1111 ± 453	5553 ± 791

**Table 2:** The number of parameters in our student networks, SAC, and PPO teacher.

Network	Ant-v3	HalfCheetah-v3
Student 6 (μ only)	16,008	9862
Student 6 (μ and σ)	16,528	10,252
SAC Teacher	98,576	73,484
PPO Teacher	23,312	142,348

**Table 3:** Architectures used for students with varying levels of compression.

Network ID	Layers	Neurons per Layer	Parameters
1	2	16	492
2	2	32	972
3	3	32	2028
4	4	32	3084
5	3	64	6092
6	4	64	10,252
7	6	64	18,572
8	4	128	36,876
9	3	256	73,484
10	4	256	139,276

**Table 4:** Average steps per second for the student sizes and various low and high-power devices.

Device	1	2	3	4	5	6	7	8	9	10
AMD Ryzen 3900X (CPU)	11,284	11,246	10,051	9112	9993	9038	7631	8794	9369	8154
Nvidia RTX 2080 Ti (GPU)	3753	3726	3322	3022	3340	3050	2559	3019	3332	3049
Nvidia A100 (GPU)	3544	3552	3247	2516	3246	2998	2609	2992	3237	2994
Nvidia GTX 1080 Ti (GPU)	1804	1813	1612	1452	1612	1453	1213	1450	1605	1448
Nvidia Jetson TX2 (CPU)	946	947	840	755	825	736	603	680	679	486
Nvidia Jetson TX2 (GPU)	285	298	271	247	270	247	210	247	270	246
Raspberry Pi 3B (CPU)	702	700	620	562	603	540	452	478	428	335

## Data Availability

The environments used in this study are part of the Gymnasium project [29] (version 2.1.0), and the pre-trained teacher models are part of the Stable Baselines3 project [31] (version 0.29.1). The code used to run the experiments is available at request from the corresponding author.

## Abbreviations

The following abbreviations are used in this manuscript:

AP	Access Point
AMR	Autonomous Mobile Robot
CPU	Central Processing Unit
DNN	Deep Neural Network
DQN	Deep Q-Network
IoT	Internet of Things
DRL	Deep Reinforcement Learning
GPU	Graphics Processing Unit
KD	Knowledge Distillation
KL	Kullback–Leibler
MSE	Mean Squared Error
PD	Policy Distillation
POMDP	Partially Observable Markov Decision Process
PPO	Proximal Policy Optimization
SAC	Soft Actor-Critic
